# Two Decades of Triazine Dendrimers

**DOI:** 10.3390/molecules26164774

**Published:** 2021-08-06

**Authors:** Eric E. Simanek

**Affiliations:** Department of Chemistry & Biochemistry, Texas Christian University, Fort Worth, TX 76129, USA; e.simanek@tcu.edu; Tel.: +1-817-257-5355

**Keywords:** dendrimer, triazine, drug delivery, gene delivery, RNA delivery, biological activity, theranostic, paclitaxel, MRI, physical organic chemistry, synthesis, membrane, separation, composite, nanoparticle, materials, atrazine, VOC

## Abstract

For two decades, methods for the synthesis and characterization of dendrimers based on [1,3,5]-triazine have been advanced by the group. Motivated by the desire to generate structural complexity on the periphery, initial efforts focused on convergent syntheses, which yielded pure materials to generation three. To obtain larger generations of dendrimers, divergent strategies were pursued using iterative reactions of monomers, sequential additions of triazine and diamines, and ultimately, macromonomers. Strategies for the incorporation of bioactive molecules using non-covalent and covalent strategies have been explored. These bioactive materials included small molecule drugs, peptides, and genetic material. In some cases, these constructs were examined in both in vitro and in vivo models with a focus on targeting prostate tumor subtypes with paclitaxel conjugates. In the materials realm, the use of triazine dendrimers anchored on solid surfaces including smectite clay, silica, mesoporous alumina, polystyrene, and others was explored for the separation of volatile organics from gas streams or the sequestration of atrazine from solution. The combination of these organics with metal nanoparticles has been probed. The goal of this review is to summarize these efforts.

## 1. Introduction

For two decades, efforts in my laboratory focused on the exploration of triazine dendrimers. When we entered the field in 2000, dendrimer science was a well-established subdiscipline that occupied the interface between small molecule chemistry and polymer science. It was guided by individuals including Donald Tomalia, Bert Meijer, George Newkome, Rene Roy, and Jean Frechet as well as the pair of Jean-Pierre Majoral and Anne-Marie Caminade. All pioneered and/or advanced classes of dendrimers that proved to be workhorses for the field including poly(ethyleneimine), poly(amidoamine), poly(esters), carbohydrates, poly(ethers), and the poly(phosphazines).

By virtue of the nature of the monomer and the chemistries employed, most dendrimers were homopolymers that could be derivatized, often statistically, on the periphery to yield function. We envisioned that exploiting the stepwise substitution of the [1,3,5]-triazine nucleus of trichlorotriazine using diamine nucleophiles offered a chance to contribute to the discipline especially in terms of the compositional diversity that might be readily generated.

Being a young, assistant professor—as well as being new to the field—Jean-Pierre Majoral, for whom this issue is dedicated, greeted me (as well as others in my cohort) with warmth and collegiality. He fully embodied the spirit of the community. He took extra efforts to make sure that we were included and heard, allowing us the opportunity to chair conference sessions and participate in other activities.

His role is perhaps best portrayed by an event that occurred while he and Anne-Marie Caminade hosted the Fifth International Dendrimers Symposium in Toulouse, France in 2007. As we watched a football match one evening after the conference proceedings had concluded, the crowds erupted in ‘Allez, allez, allez! Allez, Toulouse allez!’ The encouraging and energetic refrain of ‘Go! Go! Go!’ perfectly captured Jean-Pierre’s persona. Supportive. Enthusiastic. Optimistic. Words offered with the recognition that there was still work to be done and that we were part of an international team to do it. While my research interests have moved away from dendrimers, my appreciation for Jean-Pierre’s guiding scientific contributions to the field and his personal dedication to the development of new faculty will remain with me always. Happy birthday, Jean-Pierre! GOAL!!!

## 2. Results

This mini-review is organized in sections to discuss synthesis (convergent and divergent, with solid-supported routes discussed later), the drivers for synthesis (evaluating solubility, studying structural hypotheses), examining host–guest chemistries and biological activity, and, finally, the use of triazine dendrimers in materials science (composite materials comprising clays, silica and polymeric solid supports, and nanoparticles).

### 2.1. Synthesis

**Scheme 1:**
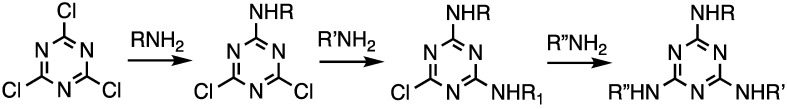
The stepwise substitution of trichlorotriazine (cyanuric chloride).

Triazine trichloride is an inexpensive building block capable of undergoing three substitution reactions in a stepwise fashion with rates that can be influenced by the choice of nucleophile, temperature, and solvent (Scheme 1).

**Scheme 2:**
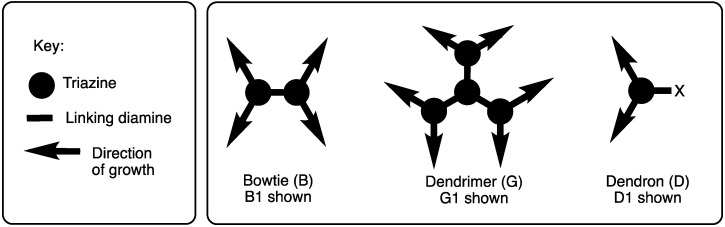
Bowtie (B), dendrimer (G), and dendron (D) architectures.

Linking triazines with diamines served as the basis for all of our efforts in this area. The discussion is subdivided into efforts that explore convergent routes and those that explore divergent routes, which is an organizational strategy that also mirrors chronology. Depending on the symmetry, dendrimers are referred to as ‘bowtie’ to convey that the central core has two branches emanating, and they are indicated as such using the letter ‘B’ (Scheme 2). In the absence of this designation, triazine dendrimers typically offer three branches (indicated with a ‘G’). The word ‘dendon’ indicates that the focus is still reactive and amenable for elaboration. Dendrons are typically the product of convergent methods. Such structures are designated with a ‘D’. The word ‘generation’ (indicated with a number that follows the designation B, G, or D) refers to the number of branching triazines that separate the core and periphery.

#### 2.1.1. Convergent Methods

Table 1 summarizes our efforts in the convergent synthesis of dendrimers.

**Scheme 3:**
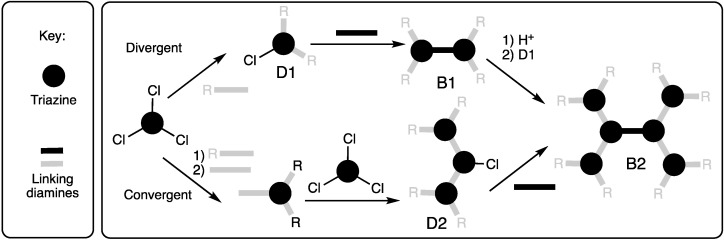
The convergent and divergent synthesis of a B2 dendrimer using *p*-aminobenzylamine (gray bar where R = BOC) and ethylene diamine (black bar).

Our first report compared the syntheses of a generation three bowtie dendrimer using either the convergent or divergent approach as shown in Scheme 3 [1]. Dr. Wen Zhang utilized *p*-aminobenzylamine (*p*-ABA) as the linking diamine due to reactivity differences of the two amines. The benzylic position was protected with BOC, and the resulting aniline was reacted with cyanuric chloride. The monochlorotriazine that results was iterated further by a selective reaction with the benzylic amine of *p*-ABA and cyanuric chloride before dimerizing with ethylenediamine to afford the bowtie. The divergent method yielded negligible amounts of product following purification with conventional silica gel chromatography.

Subsequently, he extended this convergent route to yield a generation five bowtie dendrimer which could be monitored by the iterative appearance and disappearance of the aromatic protons of the aniline intermediates [2]. Gel permeation chromatograms could be sharpened with the addition of copper (II) chloride. Yields fell off markedly at G5, which was presumably due to steric congestion at the core, leading us to propose the ‘Chemdraw rule’ within the group. That is, the limit of a convergent synthesis could be adequately predicted when the fully-extended, two-dimensional Chemdraw figure could no longer be elaborated due to overlap of the icons at the reactive core.

**Chart 1:**
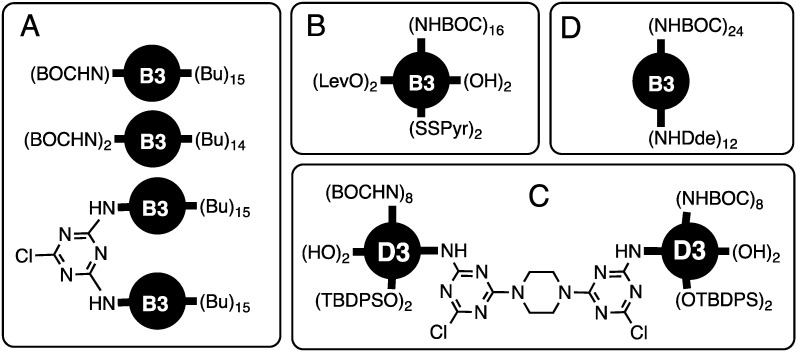
Early targets demonstrating orthogonally protective/reactive surfaces. See text for additional details. (**A**). The control the chemical composition of a dendrimer’s surface by virtue of the stepwise reactivity of trichlorotriazines coupled with a convergent synthetic route, with two targets; (**B**). The control over surface chemistry with the convergent synthesis of a dendrimer that displayed 16 BOC-protected amines, 2 hydroxyl groups, 2 TBDPS ethers, 2 levulinic amides, and 2 pyridyl disulfides; (**C**). Bowtie dendrimer retaining two reactive monochlorotriazine (MCT) groups at the core with a surface that presented 4 hydroxyl groups, 4 silyl ethers, and 16 BOC-protected amines; (**D**). Dendrimer with 12 DDE-protected amines and 24 BOC-protected amines on the surface.

With an opportunity to control the chemical composition of a dendrimer’s surface by virtue of the stepwise reactivity of trichlorotriazines coupled with a convergent synthetic route, two targets (Chart 1A), each with 16 peripheral groups, were prepared [3]. They differed by either displaying one or two BOC-protected amine groups on the periphery. Otherwise, the periphery presented *n*-butyl groups. The monosubstituted dendrimer could be deprotected and reacted with cyanuric chloride to obtain dimers that were characterized with atomic force microscopy.

The control over surface chemistry was showcased by Mr. Mackay Steffensen (PhD 2004, Texas A&M) with the convergent synthesis of a dendrimer that displayed 16 BOC-protected amines, 2 hydroxyl groups, 2 TBDPS ethers, 2 levulinic amides, and 2 pyridyl disulfides [4] (Chart 1B). This chemistry was further explored by Mr. Jongdoo Lim (PhD 2007, Texas A&M) to generate a generation three, bowtie dendrimer retaining two reactive monochlorotriazine (MCT) groups at the core with a surface that presented 4 hydroxyl groups, 4 silyl ethers, and 16 BOC-protected amines [5] (Chart 1C). Aminomethylpiperidine emerged as an amine of choice. To increase the nucleophilicity of the peripheral groups, Ms. Alona Umali (PhD 2005, Texas A&M) led an effort to prepare a similar dendrimer with 12 DDE-protected amines and 24 BOC-protected amines on the surface [6] (Chart 1D).

**Figure 1:**
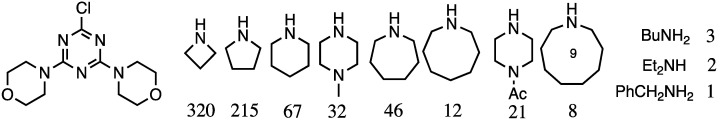
Relative reactivity of amines for a monochlorotriazine. The values for reaction with the monochlorotriazine shown are normalized to benzylamine.

In addition to controlling the composition of the periphery, Mr. Steffensen explored the nature of linking diamines [7]. The relative reactivity of a series of diamines was explored, and a 20× difference in reactivity between piperidine and primary amines was observed, which prompted broader adoption of such diamines including 4-aminomethylpiperidine and piperazine as the linking groups. This relative reactivity dataset was later expanded by Mr. Karlos Moreno (PhD 2007, Texas A&M) to yield a generation three dendrimer comprising three different, internal diamine linkers [8]. The results are summarized in Figure 1.

#### 2.1.2. Divergent Methods

When Dr. Emily Hollink joined the group, our attention had returned to divergent syntheses in spite of the poor outcome of our initial study [1]. Table 2 summarizes these efforts. We were motivated by the observation that generation three dendrimers represented a practical limit to size when using convergent synthetic routes. Divergent routes offered additional advantages. During a divergent synthesis, the number of moles of product does not change (theoretically) from the initial reaction conditions. However, in the convergent approach, the number of moles of product is reduced by integer multiples at each step as dendrons are dimerized (or otherwise multimerized) en route to the core.

**Scheme 4:**
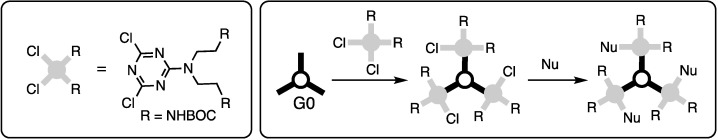
A divergent route to diversity and layered architectures. To iterate, “R” is removed, and the two-step cycle is repeated.

Using dichlorotriazines with one, two, or four BOC-protected amines, low-generation dendrimers with significant structural complexity could be prepared. That is, reaction of a tris(piperazinyl)-triazine core with a dichlorotriazine presenting two BOC groups (Scheme 4) yielded the elaborated dendrimer with three monochlorotriazines that could be additionally functionalized by taking advantage of the pendant chlorotriazine [9]. While we chose to react an amino alcohol (Nu) at each generation as proof of concept, it is unfortunate that this route to generating layers of diversity within a dendrimer structure was not more broadly adopted. The effort also represented our first use of a macromonomer to yiel two generations in one step (not shown). By employing this strategy of using a bis-BOC protected triamine with three methylene groups (instead of the two used previously) appended to a dichlorotriazine, Ms. Hannah Crampton (PhD 2008, Texas A&M) was able to achieve the synthesis of a generation five dendrimer in collaboration with Dr. Hollink [10].

This work served as the basis for our successful attempt to execute the synthesis of a generation two dendrimer at the kilogram scale. The effort, led by Dr. Abdellatif Chouai and aided by Mr. Vince Venditto (PhD 2009, Texas A&M), was ultimately checked by Mr. Brian Vanderplas of Pfizer Global Research in Groton CT who prepared both the core and monomer and chose to monitor the reactions by HPLC [11,12,13]. Mr. Venditto applied these methods and after reacting the three monochlortriazines of generation one with piperidine, he iterated to generation two and intercepted the next six monochlorotriazines on the resulting periphery with the 4-carboxypiperidine (isonipecotic acid) ester of camptothecin. After deprotection of the twelve BOC groups, the resulting amines were treated with the NHS esters of PEG-2000 to yield a final construct that was water soluble [14]. Using MCF-7 and HT-29 cell lines, he saw toxicity similar to camptothecin and greater than irinotecan. The dendrimer control showed high toxicity in HT-29 cells only.

**Chart 2:**
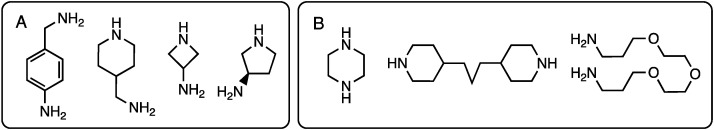
Diamines employed in the synthesis of triazine dendrimers. Diamines with selectively reactive amines are shown in panel (**A**). Symmetric diamines are shown in panel (**B**).

Recognizing solubility limitations that were arising from the use of piperazine as a linking diamine at the core of many of the dendrimers under construction, Ms. Meredith Mintzer (PhD 2009, Texas A&M) explored the use of a diamine comprising piperdine groups linked by three methylenes in the 4-positions [15]. This flexible, hydrophobic diamine (Chart 2) also precluded the existence of rotational isomers derived from the use of primary amines with hindered rotation about the triazine-N bond, thereby simplifying the NMR spectra. Dendrimers up to generation three were prepared, but these too would ultimately suffer from solubility challenges. Gas phase simulations predicted diameters of up to 3 nm.

Using the same diamine linker, Dr. Subata Patra led a team that would later elaborate this method by reacting surface amines with trichlorotriazine to yield a stable, poly(dichlorotriazine), generation four dendron that could be substituted selectively with two different amine nucleophiles, generating a diversity of surfaces [16].

**Chart 3:**
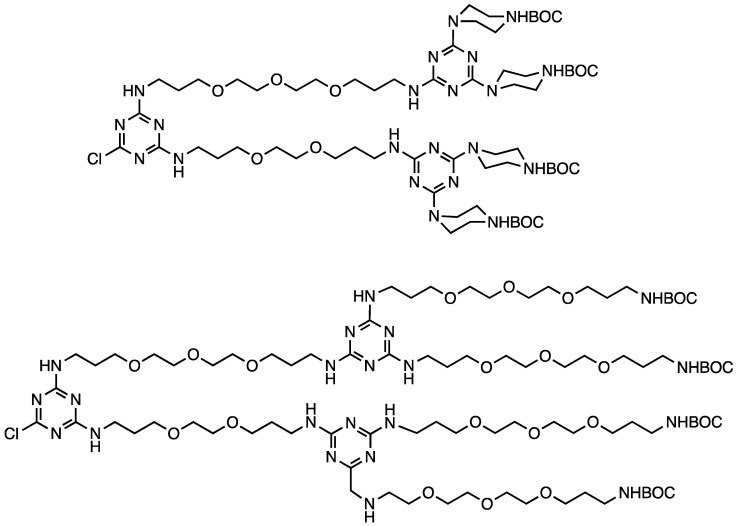
A macromonomer that yields two generations per iterative cycle during a divergent synthesis.

Initially, our divergent approaches to dendrimers yielded one generation per reaction cycle—the addition of a monochlorotriazine to an amine core followed by unmasking the BOC-protected amines that the monochlorotriazine delivered. To yield two generations per cycle, a macromonomer approach was envisioned where the two triazines (a monochlorotriazine and trisubstituted triazine bearing BOC-piperazines) would be connected with a flexible, hydrophilic linker (Chart 3). Mr. Jongdoo Lim and Ms. Meredith Mintzer were able to iterate two cycles to get to generation five materials in nine steps in 48% overall yield [17].

By substituting the reactive piperazines with the flexible linker, Dr. Lim was ultimately able to iterate out to generation 13 to yield a molecule that has dimensions of small viruses [18]. Presenting 16,384 peripheral groups (theoretically), this 8.4 MDalton dendrimer measured 30 nm in diameter by computation. These dimensions were confirmed with light scattering (Prof. O. Annunziata, Texas Christian University), atomic force microscopy (Prof. J. Maly, J.E. Purkyne University, Czech Republic), and electron microscopy (Prof. M. Kostiainen (Aalto University, Finland).

To accelerate the synthesis, Mr. Alan Enciso (PhD 2016, Texas Christian University) explored the use of microwave irradiation to arrive at a D4 dendron using an iterative convergent strategy and installed a ‘clickable’ alkyne at the core [19]. Switching to a divergent macromonomer approach wherein the protected periphery was BOC-piperazine, a generation nine dendron could be obtained in 24 h [20].

### 2.2. Drivers for Synthesis

Early drivers for synthesis focused on probing reactivity, structure, and solubility. The routes used to obtain the desired targets reflected an evolving appreciation for not only the reactivity of the linking diamines (vide supra) but also the solubility rules that were beginning to emerge. In addition to exploring solubility, the exploration of hypotheses anchored in physical organic chemistry including structure, reactivity, host/guest chemistry, and multivalency motivated target selection.

#### 2.2.1. Solubility as a Function of Surface Group and Linking Diamine

Working with Dr. Zhang, Mr. Sergio Gonzalez (PhD 2004, Texas A&M) recognized that gelation occurred when acidic chloroform was used to dissolve a G3 bowtie dendrimer with *p*-aminobenzyl amine linkers and a butyl amine surface [21]. Using a library of eight dendrimers, they found that gelation could be impacted by manipulating both the surface groups and the interior linking groups. That is, gelation could be controlled by reducing the number of hydrogen bonds (by replacing butylamine with piperidine), adding a competitive solvent (such as ethanol or DMSO), or neutralizing the organic solvent. 

During the time of the 6th International Dendrimer Symposium in Stockholm in 2009, the field was largely focused on the periphery of dendrimers rather than their interior. The reason was clear: homopolymers such as poly(propylene) imine, poly(amidoamine), and poly(arylethers) dendrimers dominated the landscape. Varying the interior was not readily possible. Herein lies another strength of triazine dendrimers.

This observation led to an evolution in our our choice of diamines: from *p*-aminobenzyl amine to 4-aminomethylpiperidine, to piperazine and ultimately to combinations that including a PEG-like diamine, 4,7,10-trioxatridecane-1,14-diamine (H_2_NCH_2_CH_2_CH_2_(OCH_2_CH_2_)_3_CH_2_NH_2_). Mr. Alan Enciso illustrated the impact of surface amines and diamine linkers in larger generation dendrimers (G2-G6), showing that cationic piperazine groups were insufficient to promote water solubility when compared with the PEG-like diamine [22]. Similarly, the use of the PEG-like diamine increased the solubility of the dendrimer in both organic solvent and aqueous solutions.

#### 2.2.2. Platforms for Physical Organic Chemistry

One hallmark of physical organic chemistry is the opportunity to perform structure–property relationships. Descriptions of dendrimers as either soft sphere and hard sphere, the limits of synthesis due to branch density, the relative mobilities of the core versus peripheral groups, and the onset of ‘dendrimer character’ were also at the forefront in Stockholm. Table 3 summarizes our work in this arena.

**Chart 4:**
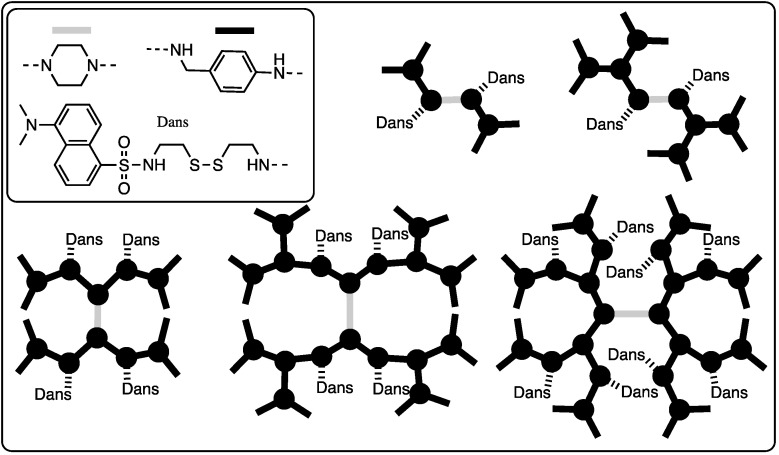
Bowtie dendrimers shown schematically with dansyl groups. Cooperative release was not observed.

The retro-Michael reactions observed within PAMAM dendrimers and the opportunity for intramolecular reactions prompted us to explore harnessing this potential by probing the opportunity for the cooperative release of disulfide-linked ligands upon exposure to a reducing agent. Dr. Zhang led a team that prepared five different bowtie dendrimers ranging in size from generation one to three [23]. The number and/or placement of disulfide-linked dansyl groups were also varied (Chart 4). Using mass spectrometry to calculate release kinetics, exchange with dithiothreitol occurs more rapidly in small dendrimers and with those where the disulfides are ‘near’ the periphery (when ‘near’ is derived from a Chemdraw depiction and not rigorous computational modeling). While cooperative exchange in the form of intramolecular reactions was not observed, the exchange process appeared to accelerate slightly as the disulfide-linked dansyl groups were cleaved off the dendrimers.

Using a similar design strategy, Dr. Zhang led a second effort to explore the rate of reduction of bowtie dendrimers ranging from generation one to four that contained disulfide cores [24]. Reduction was rapid in all cases, and quantification of differences was not possible. However, the ability of these dendrimers and the dendron precursors (a surrogate for the reduced disulfide) to solubilize pyrene increased linearly with molecular weight. Cooperative release of the guest was not observed.

**Chart 5:**
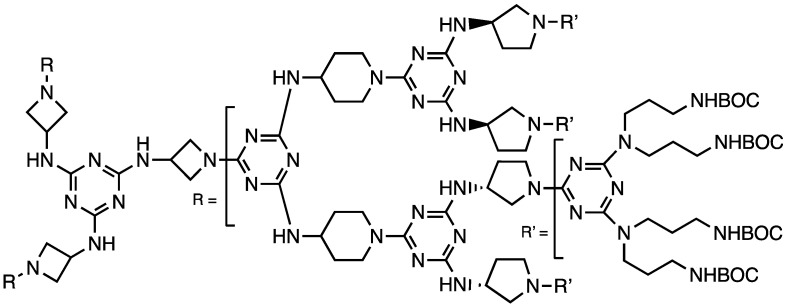
A third-generation dendrimer with different diamines at each generation used to probe conformational dynamics.

After expanding our understanding of the differential reactivity of diamines toward monochlorotriazines, Mr. Karlos Moreno synthesized a G3 dendrimer (Chart 5) with different diamine linkers at each generation starting with aminoazetidine groups at the core, then 3-aminopyrollidine groups, then 4-aminomethylpiperidine groups, and finally bis(aminopropyl)amine groups on the periphery [25]. The choice of diamine was based both on reactivity and characteristic lines in the NMR spectra to allow us to probe dynamics in different regions. NMR analysis showed solvent-dependent backfolding of the peripheral groups and solvent exclusion from the core. Relaxation analysis showed that the core moved slowly and the peripheral groups were more dynamic.

More recently, Dr. Sangram Raut under the direction of Professor Zygmunt ‘Karol’ Gryczynski (TCU) measured the intrinsic fluorescence of G1–G9 triazine dendrimers [27]. Rayleigh scattering could be used to assess aggregation, which could be disrupted by sonication and dilution. Fluorescence lifetimes, time-resolved anisotropies, and diffusional quenching indicated that the core of the dendrimer becomse increasingly compact from G1 to G5. At G7 and G9, the trend is reversed with a more porous core with extended branches and a denser periphery. Molecular dynamics performed by Professor Pavan predicted a similar trend, with most atoms close to the center of the dendrimer for G1–G5 and a shift toward the periphery in G7 and G9. We note that this change also corresponds to the point at which we observe an inversion in guest capacity. That is, Mr. Lim observed that solubilizing pyrene and camptothecin increased in the dendrimer series G1–G11 only up to G7 [26]. Capacity was reduced for both guests at G9. Professor Giovanni Pavan (SUPSI, Switzerland) used computational models to show a strong correlation to the number of internally solvating molecules of water to the guest capacity at each generation.

### 2.3. Guests and Biological Activity

The description of dendrimers as unimolecular micelles fueled our interest—and that of many others—to explore the solubilization of guests by non-covalent means. The role that multivalency could play in the delivery/presentation of bioactives also attracted design efforts. 

As previously described, the addition of Cu^2+^ to dendrimers sharpened GPC traces [2]. Pyrene served as a standard guest based on our ability to detect it.

In an early study, Dr. Zhang compared capacity as a function of dendrimer class [28]. Triazine dendrimers and poly(arylethers) accommodate 10× more pyrene (0.2 molecules/dendrimer on average) than either PAMAM or PPI dendrimers. Mixing anionic indomethacin with our cationic dendrimers led to precipitation, but anionic methotrexate could be solubilized. Both 10-hydroxycamptothecin and a bisindole methane were solubilized at four to five molecules/dendrimer. In cell culture assays, the presence of the dendrimer had no greater affect than using DMSO. Exploration of biocompatibility showed no adverse liver or kidney function upon i.p. administration up to 10 mg/kg in mice [28].

The ability of a cationic G3 dendrimer to solubilize methotrexate and mercaptopurine was explored by Mr. Michael Neerman (PhD 2006, Texas A&M Toxicology) both in vitro and in vivo in rescued mice using i.p. injection over 3 consecutive days using 10 mg/kg dendrimer (which showed toxicity equivalent to the saline control) mixed with either 2 mg/kg methotrexate or 3.5 mg/kg mercaptopurine [29]. In both cases, premixing with dendrimer reduced the hepatotoxicity of the drug as measured by alanine transaminase levels.

While the inversion of guest capacity (both pyrene and camptothecin) in higher-generation dendrimers was already discussed [26], we note that the maximum capacity of camptothecin was 8.3 molecules/dendrimer at G7 (517 kDa). The data also suggested that using non-covalent association of drugs in these dendrimers could be limiting: at G7, the loading is 0.6% by weight at G7, although the relationship between structure and capacity still demands exploration.

By covalently conjugated the drug, six molecules could be appended to a G2 dendrimer [14], leading to loadings of 7–40 wt% depending on the degree of PEGylation. In cell culture assays (MCF7, HT29), the cationic dendrimer and one PEGylated (PEG 2000) up to 12 times showed toxicity similar to camptothecin (IC_50_ = 0.2 uM) and approximately 10× better than irinotecan (IC_50_ = 33–52 uM) with a range of IC_50_ values of 0.5–2.2 uM depending on the cell line.

Ms. Meredith Mintzer collaborated with groups led by Professor Thomas Kissel (Marburg) and Dr. Olivia Merkel (Marburg) to explore DNA and RNA transfection in a series of studies that were complemented by computational models provided by Professor Giovanni Pavan (SUPSI) [30,31,32,33,34].

**Chart 6:**
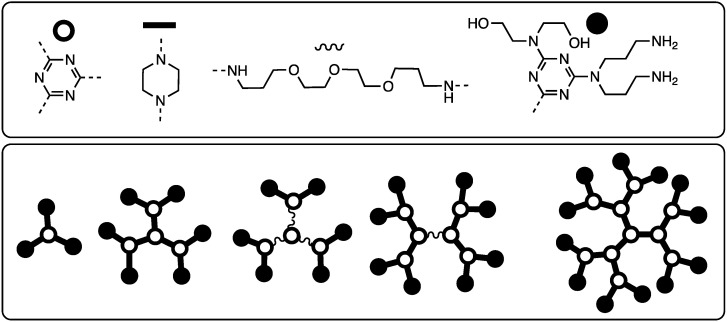
A series of rigid and flexible dendrimers for transfection [31].

In the first study focused on gene delivery, G1–G3 and B2 dendrimers with linking diamines chosen to vary flexibility provided between six and 24 cationic amines and equivalent number of hydroxyls (dependent on generation) [30]. These materials (Chart 6) were characterized by a host of methods including DNA condensation efficiency, size, surface charge, morphology, and toxicity including hemolysis. Using L929 and MeWo cells, the flexible G2 dendrimer displayed a higher transfection efficiency than the industry standards, Superfect or 25 kDa poly(ethylene imine) while displaying lower toxicity. Professor Pavan used computation to explore the basis for the differences observed between flexible and rigid dendrimers to examine different modes of binding [31].

Subsequently, nine dendrimers varying in generation (one to three), shape (bowtie or traditional), and surface functionality (number of amine, hydroxyl and hydrophobic groups) were examined for the knockdown efficiency of a luciferase reporter gene in HeLa/Luc cells [32]. Transfection efficiency was greatly affected by structure with rigid G2 dendrimers performing the best. Employing guanidinium groups as the cation with hydrophobic auxiliary groups improved performance over primary amines with hydrophilic groups. Upon i.v. administration, the dendriplexes accumulated in the alveolar cells of the lungs. This work provided a foundation for contextual comparison and reviews [33,34].

In work inspired by the creation of DNA-based materials, Dr. Steven Bell and Ms. Megan McLean (PhD 2004, Texas A&M) installed a DNA oligonucleotide onto G2 and G3 dendrons using thiol-disulfide exchange at the focus, periphery, or both [35].

The use of thiol-disulfide exchange using dendrons and bowtie dendrimers bearing pyridyl disulfide groups was also explored by Ms. Alona Umali, who prepared a generation three dendron and its dimer, a generation three bowtie bearing either four or eight pyridyl disulfide groups, respectively [36]. The small molecule, thiol-containing drug, captropril, could be installed quantitatively. A peptide with a cystine residue at its N-terminus, CLKKDDRA, could also be installed, but the use of guanidinium hydrochloride proved critical in order to obtain hexavalent constructs. Sterics and charge are believed to be the primary factors preventing the realization of the octavalent display.

**Chart 7:**
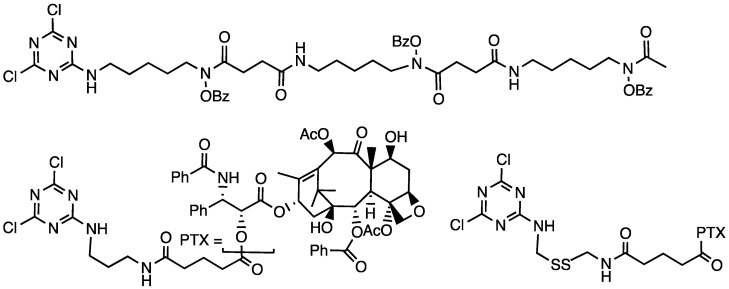
A bioactive conjugated to a dichlorotriazine for installation on an amine-terminated dendrimer. Both protected desferrioxamine B (top) and paclitaxel (bottom) have been incorporated into dendrimers.

Using the G2 dendrimer available at the kilogram scale, Mr. Jongdoo Lim and Mr. Vince Venditto installed the iron chelator desferrioxamine B (in protected form, Chart 7) as a dichlorotriazine and reacted with 12 primary amines presented on the dendrimer periphery [37]. The poly(monochlorotriazine) that resulted was ‘capped’ with 4-hydroxylmethylpiperidine, leading to a construct that was 56 wt% drug. Spectrophotometric analysis showed that all 12 DFO groups could chelate iron.

To explore the potential of triazine constructs to serve as antibacterials, Mr. Raghavendra Sreeperumbuduru (MS 2015, TCU) prepared 18 constructs that varied in valency (2, 3, 4, or 6) and alkylation of the periperal DABCO groups (methyl, benzyl, or dodecyl) [38]. These materials were evaluated against *Staphylococcus aureus* (Newman), methicillin-resistant *S. aureus* (MRSA; Sanger 252), and *Escherichia coli* (K-12). Antibacterial activity was influenced both by the alkylating group (dodecyl > benzyl > methyl) and valency with divalent and trivalent compounds outperforming tetravalent and hexavalent molecules.

As part of an international team focused on the production of anti-sepsis agents, triazine dendrimers were explored as surrogates for PAMAM dendrimers to determine whether variability and stability challenges encountered using half-generation PAMAM dendrimers could be alleviated. The goal was to *N*-acylate glucosamine groups with carboxylic acids on the dendrimer periphery to interfere with the complexation of lipopolysaccharide with TLR4 and MD-2 on cell surfaces. The kilogram-scale G2 dendrimer presenting 12 surface amines was used as a starting material by Dr. Sanjiv Lalwani. Conversion to tertiary amines with methyl bromoacetate followed by hydrolysis yielded 24 carboxylic acids. Electrophoretic analysis revealed a similar pI to the target PAMAM [39]. Capillary electrophoresis provided additional insights into the purity of the target and PAMAM materials [40]. Computation was used to guide the design and rationalize early biological activities [41].

However, by far, our primary focus in the generation of bioactive constructs for translational exploration focused on covalently incorporating paclitaxel into dendrimers (Chart 7), which was described in a series of manuscripts by Dr. Lim and ultimately evaluated by Dr. Xiankai Sun and coworkers at the UT Southwestern Medical Center.

To evaluate the extent of drug loading in these potential theranostics, Mr. Lim executed a convergent synthesis to yield a generation three bowtie with 2 phenols at the core for radioiodination, 12 PEG groups, and 16 ester-linked paclitaxel groups that were incorporated through reaction between the amine-terminated dendrimers and a paclitaxel-derivatized dichlorotriazine [42]. PEGylation occurred last by reacting the poly(monochlorotriazine) dendrimer with a diamine and NHS-PEG (with either 2 kDa or 5 kDa PEG). The targets are obtained in nine steps in yields of 50%. Using 2 kDa PEG, the construct is 46 kDa, 30 wt% paclitaxel, 52 wt% PEG, and 18 wt% dendrimer. Using 5 kDa PEG, the constuct is 77 kDa, 18 wt% paclitaxel, 71 wt% PEG, and 11 wt% dendrimer. Water solubility with high drug loading had been accomplished.

In collaboration with Dr. Anil Patri and his team at the Nanotechnology Characterization Laboratory (NCL) at Fort Dettrick, Maryland, a more thorough characterization assessment was performed [43]. The NCL found that the size measured for the construct was dependent on the technique employed. Mass spectrometry confirmed that the molecular weight of the molecule was 40 kDa. However, light scattering suggested an aggregate with a molecular weight of 400 kDa based on a hydrodynamic radius of 15–20 nm.

HPLC was employed to assess the purity and release kinetics of paclitaxel. Release was insignificant in organic solvent and in aqueous solutions at neutral or low pH. In vitro experiments with plasma obtained from humans, rats, or mice showed that release was non-linear and ranged from 7 to 20% over a 48 h period. In cell culture experiments with HepG2 (hepatocarcinoma), LLC-PK1 (porcine renal proximal tubule), and LS174T (human colon carcinoma), the construct was two to three orders of magnitude less toxic than paclitaxel. However, it showed similar cytotoxicity to Abraxane in L174T cells.

Similar to paclitaxel, the dendrimer induced caspase 3-dependent apoptosis. It was not hemolytic and did not induce platelet aggregation in vitro, but it did reduce collagen-induced platelet aggregation in vitro. The complement system was activated in vitro, which was most likely due to trace amounts of free paclitaxel.

Biodistribution was evaluated in SCID mice bearing PC3 tumors. Tumor localization occurred for all three dendrimers. One construct showed a tumor/blood ratio of 1.2:1 and a tumor to muscle ratio of 24:1. Renal excretion was observed (35–55%). These results compelled us to look to efficacy studies.

Upon administering the dendrimer or Abraxane once per week for three weeks at doses of 10, 25, 50, and 100 mg paclitaxel/kg in non-tumored athymic nude mice, similar in vivo toxicities were observed. Both were nontoxic at these doses. The arrest of tumor growth was observed in a prostate tumor model (PC-3-h-luc) over 70 days.

To reduce the dose necessary for efficacy and enhance cleavage rates, the tether connecting paclitaxel to the dendrimer was modified (Chart 7) to include a disulfide bond in addition to the ester-linked drug [44]. Cell-based assays proved compelling. At 72 h incubation, the IC_50_ of free paclitaxel was 4.5 nM. The dendrimer comprising only an ester linkage had an IC_50_ of 29 nM regardless of whether it was coadministered with dithiothreitol or glutathione. In the absence of reductant, the disulfide-containing constructs had IC_50_ values of ≈74 nM, which dropped when reductant was added to 26 nM (1 mM glutathione) or 13 nM (1 mM DTT).

Five BALB-C mice were given three i.v. doses of one of the disulfide-linked dendrimers at 60 mg/kg (based on paclitaxel) on days 0, 4, and 8. This dose corresponds to 3× the maximum tolerated dose of paclitaxel. No adverse effects were observed over three weeks. Visual inspection of organs and blood levels of alanine transaminase or urea showed no deviations from normal. A Bolton–Hunter group was installed for radioiodination. The values recorded for half-lives were similar: t_1/2a_ ≈0.4 h; t_1/2b_ 15–20 h. The highest accumulation was observed in the liver and spleen.

The hypothesis that toxicity would be increased was borne out in vitro and in vivo. Intravenous administration was performed weekly for either one, two, or three weeks at doses that varied from 50 to 200 mg paclitaxel/kg [45]. Tumor arrest and regression was observed over the 10-week treatment period without mortality at 50 mg paclitaxel/kg administered thrice. A ‘cure’ was achieved, but the dosing was competitive with Abraxane, and it was not greatly enhanced as was desired.

Our efforts moved to the preparation of a next-generation dendrimer designed to reduce the number of PEG chains from 12 to eight in order to increase the homogeneity within the sample, to include a single phenol for radioiodination at the focus, and to increase the number of paclitaxel groups from 12 to 16 [46]. Dr. Changsuk Lee led the synthetic efforts which installed all paclitaxel groups and 5–8 5kDa PEG chains leading to a construct that was 63 wt% PEG, 22 wt% paclitaxel, and 15% triazine dendrimer.

Paclitaxel was released slowly in mouse and rat plasma (8%). SPECT/CT imaging was used to follow biodistribution and tumor uptake. The elimination half-life was 2.7 h, and the distribution half-life was 32 h. This target persists in the vasculature longer than previous ones and showed high tumor uptake as well as low uptake in the lung, liver, and spleen. Tumor saturation studies using PC3 tumors revealed that saturation occurs at a dose between 32 and 70 mg paclitaxel/kg.

**Chart 8:**
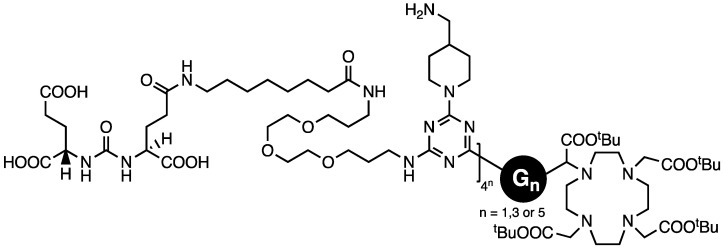
The targeting ligand DUPA was installed as a dichlorotriazine, and the resulting species was reacted with aminomethylpiperidine prior to deprotection.

We concluded that triazine dendrimers offer a promising platform for drug delivery with a high degree of control that can be exercised over composition. However, targeting that relied wholly on the enhanced permeation and retention that tumors show toward macromolecules appeared too insufficient to garner clinical advantage. Accordingly, our final study addressed active targeting using PSMA ligands, DUPA, on generation one, three, and five dendrons afforded by a divergent macromonomer synthesis wherein the diamines comprised reactive piperazines and flexible 4,7,10-trioxododecanediamine [47]. Dr. Lim installed DUPA by appending it to a dichlorotriazine leading to materials with 4, 16, or 64 ligands (Chart 8). A DOTA group was installed at the focus. The constructs maintained a pendant amine for the subsequent installation of an agent such as paclitaxel and PEG.

Assays using PSMA-positive (PC3-PIP) and PSMA-negative (PC3-FLU) cells showed that the D1 construct had the highest PSMA-(+):PSMA-(−) ratio of 10:1. The largest dendron showed 12× higher binding affinity (IC_50_ 23.6 nM) to PSMA-(+) cells than the D1 construct. However, these large molecules also showed high non-specific binding to PSMA-(−) cells.

Using SCID mice with a PSMA-(+) and PSMA-(−) tumor on each shoulder, the D3 construct showed the lowest tumor uptake. The D1 construct showed the best tumor/muscle and PSMA-(+)/PSMA-(−) ratios. The D5 construct showed similar levels of uptake in both tumors, supporting the significant role of the EPR effect.

In summary, triazine dendrimers are well-poised for consideration for future studies. In addition to the promising activities observed, our understanding of how the PEGylation strategy influences biodistribution [48] and additional biodistribution studies using these dendrimers as a host for MRI imaging agents [49,50] help to guide design. In vitro and in vivo studies complement these efforts, including studies of maximum tolerated dosing [51], platelet aggregation [52], complement activation [53], and potential antigenicity [54].

Table 4 identifies guests that have been explored as well as whether their association has been covalent or non-covalent.

### 2.4. Applications in Materials Science

We (and others) recognized early on that triazine dendrimers need not be limited to biomedical applications. The pursuit of these molecules for application in materials science is summarized in Table 5.

#### Membranes

The ease of triazine dendrimer synthesis led to their exploration as coatings on solid membranes in collaboration with Professors David Ford and Dan Shantz of the Department of Chemical Engineering at Texas A&M University [55,56,57,58,59].

Mr. Erick Acosta (PhD 2003, Texas A&M) focused initial efforts on mesoporous silica (SBA-15) wherein an amine-coated surface offered the opportunity to execute simple synthetic steps by submersion in solutions containing cyanuric chloride and aminomethylpiperidine in an iterative fashion, leading to generation four dendrons grafted on the surface [55]. Thermal gravimetric analysis, transmission electron microscopy, adsorption isotherms, and mass spectrometry were used to corroborate the structure.

This work was elaborated with Mr. Sergio Gonzalez offering synthetic support in order to explore the affect that the choice of linking diamine might have on synthesis and application [56]. Aminomethylpiperidine was substituted with piperazine as well as trimethylbispiperidine to explore the role of flexibility and rigidity with an eye toward controlling porosity within the organic layer. Characterization relied on similar strategies as described above. The work established that porosity could be tuned independently with either the size and composition of the dendrimer.

Mr. Sergio Gonzalez also assisted the Ford group in applying these methods to alumina membranes, allowing triazines to be compared with alkylsilanes including phenyl and octadecyl derivatives [56]. The triazine materials provided the separation of toluene from N_2_ with efficiencies similar to the octadecylsilane-derived materials and superior to that of phenylsilane-derived materials. Moreover, these materials showed similar separation factors compared with polymer membranes, but faster separation rates.

Using a similar strategy, reverse-selective membranes were prepared and evaluated on mesoporous ceramic supports. Propane removal from N_2_ is achieved by incorporating hydrophobic dodecyl chains into generation one to three dendrons anchored with an aminopropyldimethylsilane layer [57].

The method developed could also be applied to other solid supports including silica gels [58]. Both divergent and convergent approaches were employed to functionalize surfaces with piperazine-linked triazine dendrons. For the convergent approach, the dendron with a monochlorotriazine core was reacted with the piperazine-coated surface. Iterative reactions with cyanuric chloride and piperazine were performed for the divergent approach. A uniformed 20% *w*/*w* loading was observed when the convergent method was employed, regardless of dendron added. Increasing amounts of material were observed at each generation using the divergent approach, but mass spectrometry of the HF-treated materials revealed structural defects. The divergent materials were successful at sequestering more atrazine hypothetically through covalent reactions with this monochlorotriazine.

The sequestration of atrazine has also been pursued using piperidine-functionalized poly(N-isopropylacrylamide) by taking advantage of its LCST properties [59]. The covalent capture of atrazine has been pursued using nucleophilic polystrene resins derivatized with isonipecotic acid (4-carboxypiperidine) [60] and similarly on Janda Gels of different mesh sizes [61].

The use of solid supports for the solid phase synthesis of dendrons did not escape our attention. Dr. Adela Huang of Kaohsiung University developed a method that yielded generation one to four dendrons by the iterative reaction of a cyanuric chloride and aminomethylpiperidine from a Rink Amide resin [62]. The Kaiser test revealed the presence of unreacted amines during the cyanuric chloride addition step. Treatment of beads with Alizarin R, a dye that is reactive with dichlorotriazines, indicated whether amine addition was complete.

Smectite clay was probed as a solid support using dendrons of generation one to three as well as a linear G2 analogue [63]. Mr. Acosta found that the generation one and the linear generation two analogue appear to intercalate with the gallery spacing increasing from 1.47 nm to 3 nm or 4.6 nm, respectively. Generation two and three dendrons do not lead to significant interlamellar distances: spacing increases from 1.47 to 1.57 nm. When these clays are treated with a surfactant that retains a reactive secondary amine, atrazine sequestration is observed [64].

More recently, the role of triazine dendrimers in synthesizing or stabilizing nanoparticles has been explored [65,66]. In collaboration with Professor Ahmed Mohammed (Sharjah University, UAE), generation one to three dendrons with a focus containing a tetrachloroaurate diazonium salt yielded nanoparticles when treated with a reducing agent. The particles appear to display a Au-C bond and are markedly robust and stable in solution for longer than a year. The size and dispersity of the nanoparticles decrease with increasing the generation of dendrons with a shift from ≈2–18 nm at D1 to 4–12 nm at D2 and to 4–8 nm at D3. The systems have been modeled by Professor Pavan’s group [65].

Mr. Fermin Ramirez-Crescencio, a visitor from UNAM, Mexico, determined that gold nanoparticles could be synthesized within acetylated G5 dendrimers (which show LCST behavior) after titration with metal ions. Preliminary observation suggests that other nanoparticles could be accessed in a similar manner including those derived from copper, platinum, palladium, and iridium. Iridium and platinum nanoparticle encapsulated dendrimers also were obtained by mechanically milling the metallic precursors in the presence of sodium borohydride and the triazine dendrimers [67].

The use of a triazine dendrimer in a gluscose-sensing device was also pursued [68]. In collaboration with Professors Cote and Pishko of the Departments of Biomedical and Chemical engineering at Texas A&M, an optical device relying on a generation two glycosylated dendrimer and its binding to Concanavillin A was created.

## 3. Conclusions

While our 20 years of triazine dendrimer chemistry have been reviewed in the past [70,71,72,73,74,75,76,77], this review is the first attempt to catalogue our entire effort. With the exception of the methodology developed for installing NH_2_ groups on triazines with benzylated amines [78], the use of cyanuric chloride to generate hyperbranched PPI [79], the use of these materials as objects for light-driven transportation [80], and their use as a lens to view mechanistic organic chemistry [81], this effort summarizes the entirety of our body of work, which was performed by a host of talented coworkers and collaborators from my time at Texas A&M University and concluding at Texas Christian University. Throughout, we looked to the community for lessons learned and inspiration. The work from the labs of Jean-Pierre Majoral and Anne-Marie Caminade had a profound influence on what we did and how we could do it. Once again, and with gratitude, happy 80th birthday Jean-Marie!

## Figures and Tables

**Table 1:** Synthetic efforts utilizing the convergent strategy. ‘B’ refers to a two-branched bowtie dendrimer, ‘G’ refers to a three-branched dendrimer, ‘D’ refers to a dendron with a reactive focal point. The number following these letters corresponds to the generation. *p*-ABA is *p*-aminobenzlamine, common protecting groups incorporated on the surface include BOC, TBDPS, pyridyl disulfides (PyrSS), levulinic amides (levul), or DDE groups. MCT is monochlorotriazine. OH indicates free hydroxyls.

Focus	Style	Notes	Ref #
Convergent v. divergent methods	B3	First report, orthogonal linker (*p*-ABA)	[1]
Convergent, GPC analysis	D5	*p*-ABA, Cu^2+^ sequestration	[2]
One or two reactive sites of 16 total	B3	Unique sites, dimers made, computation	[3]
Multiple protected peripheral groups	B3	16 BOC, 2 OH, 2 TBDPS, 2 levul, 2PyrSS	[4]
Multiple protected peripheral groups	B3	16 BOC, 4 OH, 4 TBPDS, 2-MCT	[5]
Two protected amines on surface	G3	24 BOC, 12 DDE	[6]
Varied internal diamines	D3	Relative reactivity data	[7]
Larger survey of amine reactivity	G3	Three different diamines	[8]

**Table 2:** Synthetic efforts using the divergent strategy.

Focus	Style	Notes	Ref #
Convergent v. divergent methods	B3	Poor yields	[1]
Efficient divergent synthesis	G2,3	Opportunity for layer-by-layer diversity	[9]
BisBOC-dichlorotriazine	G5	Choice of bisBOC triamine affords big	[10]
Kilogram scale monomer	-	Checked by *Org. Syn.*	[11]
Kilogram scale dendrimer	G2	Checked by *Org. Syn.*	[12]
Full report kg scale	G2	Complete synthesis	[13]
Interception G2 w/bioactive	G2	Installed 6 camptothecin and 12 PEGs	[14]
Trimethylenebispiperidine	G3	Computation predicts 3 nm dia.	[15]
Reaction with trichlorotriazine	D4	Solution synthesis, different capping amines	[16]
Macromonomer routes	G5	Two generations/iteration	[17]
Synthesis of a ‘virus-sized’ dendrimer	G13	Macromonomer w/flexible hydrophilic linker	[18]
Rapid, microwave synthesis	D4	Iterative, convergent w/flexible diamine	[19]

**Table 3:** Inquiries from physical organic chemistry.

Focus	Gn	Notes	Ref #
Intramolecular (interbranch) reactivity	G1-G3	Disulfide-linked cleavage not cooperative	[23]
Steric protection of the core	B1-B4	Disulfide core cleavage rates	[24]
Conformational dynamics	G5	NMR studies reveal dynamics	[25]
Guest capacity as a function of generation	G1-G11	Inversion of capacity in large dendrimers	[26]
Internal, intermolecular interactions	G1-G9	Fluorescence studies suggest	[27]

**Table 4:** Guests and ligands explored.

Guest/Ligand	Cov/Non	Gn	Notes	Ref #
Cu(II)	Non	D1–D5	Reduction of aggregation by GPC	[2]
Pyrene	“”	G1–G3	Similar to polyethers, 10× than PPI/PAMAM	[28]
“”	“”	B1–B4	Guest concentration correlates with MW	[24]
“”	“”	G1–G11	Inversion of guest capacity at high generation	[26]
Methotrexate	“”	G1–G3	Solubilization study & reduced hepatotoxicity	[28]
“”	“”	G3	3 molecules/dendrimer solubilized	[29]
Indomethacin	“”	G1–G3	Precipitates with cationic dendrimer	[28]
10-Hydroxycamptothecin	“”	G1–G3	4–5 molecules/dendrimer	[28]
Bis-indole methane	“”	G1–G3	4–5 molecules/dendrimer	[28]
Mercaptopurine	“”	G3	16 molecules/dendrimer	[29]
Camptothecin	Cov	G2	6 molecules/dendrimer,	[14]
“”	Non	G1–G9	Inversion of capacity at G7	[26]
DNA	“”	G1–G3, B2	Flexible G2 outperforms SuperFect, PPI	[30]
DNa/siRNA	“”	G1–G3, B2	Computational models of flexible v. rigid	[31]
RNAi	“”	G1–G3, B2	In vitro and in vivo analysis with SAR	[32]
DNA	Cov	D2, D3	Disulfide linked, 12 base oligos	[35]
Captopril	“”	G3, D3	Disulfide linked tetra- and octavalent	[36]
CLKKDDRA peptide	“”	“”	Disulfide linked tetra- and octavalent	[36]
Desferrioxamine	“”	G3	56 wt% drug	[37]
DABCO	“”	D1, B1, G1	Divalent–hexavalent displays, antibacterial	[38]
*N*-acyl glucosamine	“”	G2	Anti-sepsis candidate model	[41]
Paclitaxel	“”	B3	Synthesis (con), 16 PTX, 12 PEG, radiolabel	[42]
“”	“”	G3	Ester-linked drug, broad assessment	[43]
“”	“”	G3	Ester/disulfide biodistribution	[44]
“”	“”	G3	“Cures” observed with disulfide in linker	[45]
“”	“”	D4	Synthesis/evaluation of homogeneous target	[46]
DUPA	“”	D1, D3, D5	PSMA-(+) tumors targeted	[47]
PEG	“”	Varied	PEGylation strategy influences biodistribution	[48]
DOTA/DPTA	“”	G3, G5	Biodistribution of Gd-MRI agents	[49]
DOTA	“”	D2	DOTA at focus, maleimides on periphery	[50]

**Table 5:** Solid supports and triazine dendrimers.

Support	Gn	Notes	Ref #
Mesoporous Si (SBA-15)	D1-D4	Synthetic methods; iterative C_3_N_3_Cl_3_ and diamine	[55]
“”	D1-D3	Role of linking diamine, porosity control	[56]
Mesoporous alumina	Hyper	Good separation factors, faster than polymer membranes	[57]
Ceramic	D1-D3	Good selectivity for VOCs	[58]
Silica	D1-D3	Convergent and divergent synthesis	[59]
LCST polymers	D1	Solid phase extraction with thermoresponsive polymers	[60]
Polystyrene	NA	Piperidine functionalized supports sequester atrazine	[61]
Janda gel	NA	Atrazine sequestration with different mesh resins	[62]
Solid phase synthesis	D1-D3	SPPS methods applied to dendrimer synthesis	[63]
Smectite clay	D1-D3	Frustrated intercalation for D2, D3, not D1 or linear D2	[64]
“”	NA	Reactive surfactant sequesters atrazine	[65]
Gold nanoparticles	D1-D3	Synthesis w/ dendritic surfactant w/diazoninum focus	[66]
“”	G5	Au nanoparticles described, others (Cu, Pd, Ir, Pt) observed	[67]
Iridium nanoparticles	D1-D3	Obtained by mechanical milling	[68]
Glucose-sensing device	G3	Optical measurements using glycosylated dendrimer	[69]

## Data Availability

Data provided in the cited references or supporting information associated with each manuscript.

## References

[1] Zhang W., Simanek E.E. (2000). Dendrimers based on melamine. Divergent and orthogonal, convergent syntheses of a G3 dendrimer. Org. Lett..

[2] Zhang W., Simanek E.E. (2001). Synthesis and characterization of higher generation dendrons based on melamine using *p*-aminobenzylamine. Evidence for molecular recognition of Cu(II). Tetrahedron Lett..

[3] Zhang W., Nowlan D.T., Thomson L.M., Lackowski W.M., Simanek E.E. (2001). Orthogonal, convergent syntheses of dendrimers based on melamine with one or two unique surface sites for manipulation. J. Am. Chem. Soc..

[4] Steffensen M.B., Simanek E.E. (2004). Synthesis and manipulation of orthogonally protected dendrimers: Building blocks for library synthesis. Angew. Chem. Int. Ed..

[5] Lim J., Simanek E.E. (2005). Toward the next-generation drug delivery vehicle: Synthesis of a dendrimer with four orthogonally reactive groups. Mol. Pharm..

[6] Umali A.P., Crampton H.L., Simanek E.E. (2007). Triazine dendrimers with orthogonally protected amines on the periphery. Masking amines with Dde and BOC groups provides an alternative to carrying protected alcohols and disulfides through an iterative synthesis. J. Org. Chem..

[7] Steffensen M.B., Simanek E.E. (2003). Chemoselective building blocks for dendrimers from relative reactivity data. Org. Lett..

[8] Moreno K.X., Simanek E.E. (2008). Identification of diamine linkers with differing reactivity and their application in the synthesis of melamine dendrimers. Tetrahedron Lett..

[9] Hollink E., Simanek E.E. (2006). A divergent route to diversity in macromolecules. Org. Lett..

[10] Crampton H., Hollink E., Perez L.M., Simanek E.E. (2007). A divergent route towards single-chemical entity triazine dendrimers with opportunities for structural diversity. New J. Chem..

[11] Chouai A., Venditto V.J., Simanek E.E. (2009). Synthesis of 2-[3,3′]-Di-(tert-butoxycarbonyl)-aminoproplamine]-4,6-dichloro-1,3,5-triazine as a monomer and 1,3,5-[tris-piperazine0-triazine as a core for the large scale synthesis of melamine (triazine) dendrimers. Org. Syn..

[12] Chouai A., Venditto V.J., Simanek E.E. (2009). Large scale synthesis of a generation-1 melamine (triazine) dendrimer. Org. Syn..

[13] Chouai A., Simanek E.E. (2008). Kilogram-scale synthesis of a second-generation dendrimer based on 1,3,5-triazine using green and industrially compatible methods with a single chromatographic step. J. Org. Chem..

[14] Venditto V.J., Allred K., Allred C.D., Simanek E.E. (2009). Intercepting the synthesis of triazine dendrimers with nucleophilic pharmacophores: A general strategy toward drug delivery vehicles. Chem. Comm..

[15] Mintzer M.A., Perez L.M., Simanek E.E. (2010). Divergent synthesis of triazine dendrimers using a trimethylene-dipiperidine linker that increases efficiency, simplifies analysis, and improves product solubility. Tetrahedron Lett..

[16] Patra S., Kozura B., Huang A.Y., Enciso A.E., Sun X., Hsieh J.T., Kao C.L., Chen H.T., Simanek E.E. (2013). Dendrimers Terminated with Dichlorotriazine Groups Provide a Route to Compositional Diversity. Org. Lett..

[17] Lim J., Mintzer M.A., Perez L.M., Simanek E.E. (2010). Synthesis of Odd Generation Triazine Dendrimers Using a Divergent, Macromonomer Approach. Org. Lett..

[18] Lim J., Kostiainen M., Maly J., da Costa V.C., Annunziata O., Pavan G.M., Simanek E.E. (2013). Synthesis of Large Dendrimers with the Dimensions of Small Viruses. J. Am. Chem. Soc..

[19] Enciso A.E., Abid Z.M., Simanek E.E. (2014). Rapid, semi-automated convergent synthesis of low generation triazine dendrimers using microwave assisted reactions. Polym. Chem..

[20] Enciso A.E., Ramirez-Crescencio F., Zeiser M., Redón R., Simanek E.E. (2015). Accelerated synthesis of large generation triazine dendrimers using microwave assisted reactions: A 24 h challenge. Polym. Chem..

[21] Zhang W., Gonzalez S.O., Simanek E.E. (2002). Structure-activity relationships in dendrimers, based on triazines: Gelation depends on choice of linking and surface groups. Macromolecules.

[22] Enciso A.E., Garzoni M., Pavan G.M., Simanek E.E. (2015). Influence of linker groups on the solubility of triazine dendrimers. New J. Chem..

[23] Zhang W., Tichy S.E., Pérez L.M., Maria G.C., Lindahl P.A., Simanek E.E. (2003). Evaluation of multivalent dendrimers based on melamine: Kinetics of thiol-disulfide exchange depends on the structure of the dendrimer. J. Am. Chem. Soc..

[24] Zhang W., Lalwani S., Chouai A., Simanek E.E. (2009). Synthesis and Characterization of Anionic Triazine Dendrimers with a Labile Disulfide Core. Isr. J. Chem..

[25] Moreno K.X., Simanek E.E. (2008). Conformational analysis of triazine dendrimers: Using NMR spectroscopy to probe the choreography of a dendrimer’s dance. Macromolecules.

[26] Lim J., Pavan G.M., Annunziata O., Simanek E.E. (2012). Experimental and Computational Evidence for an Inversion in Guest Capacity in High-Generation Triazine Dendrimer Hosts. J. Am. Chem. Soc..

[27] Raut S., Enciso A.E., Pavan G.M., Lee C., Yepremyan A., Donald A., Tomalia D.A., Simanek E.E., Gryczynski Z. (2017). Intrinsic Fluorescence of Triazine Dendrimers Provides a New Approach to Study Dendrimer Structure and Conformational Dynamics. J. Phys. Chem. C.

[28] Zhang W., Jiang J., Qin C., Pérez L.M., Parrish A.R., Safe S.H., Simanek E.E. (2003). Triazine dendrimers for drug delivery: Evaluation of solubilization properties, activity in cell culture, and in vivo toxicity of a candidate vehicle. Supramol. Chem..

[29] Neerman M.F., Chen H.-T., Parrish A.R., Simanek E.E. (2004). Reduction of drug toxicity using dendrimers based on melamine. Mol. Pharm..

[30] Merkel O.M., Zheng M., Mintzer M.A., Pavan G.M., Librizzi D., Maly M., Höffken H., Danani A., Simanek E.E., Kissel T. (2011). Molecular modeling and in vivo imaging can identify successful flexible triazine dendrimer-based siRNA delivery systems. J. Control. Release.

[31] Merkel O.M., Mintzer M.A., Librizzi D., Samsonova O., Dicke T., Sproat B., Garn H., Barth P.J., Simanek E.E., Kissel T. (2010). Triazine Dendrimers as Nonviral Vectors for in Vitro and in Vivo RNAi: The Effects of Peripheral Groups and Core Structure on Biological Activity. Mol. Pharm..

[32] Pavan G.M., Mintzer M.A., Simanek E.E., Merkel O.M., Kissel T., Danani A. (2010). Computational Insights into the Interactions between DNA and siRNA with “Rigid” and “Flexible” Triazine Dendrimers. Biomacromolecules.

[33] Merkel O.M., Mintzer M.A., Sitterberg J., Bakowsky U., Simanek E.E., Kissel T. (2009). Triazine Dendrimers as Nonviral Gene Delivery Systems: Effects of Molecular Structure on Biological Activity. Bioconj. Chem..

[34] Mintzer M.A., Merkel O.M., Kissel T., Simanek E.E. (2009). Polycationic triazine-based dendrimers: Effect of peripheral groups on transfection efficiency. New J. Chem..

[35] Bell S.A., McLean M.E., Oh S.K., Tichy S.E., Zhang W., Corn R.M., Crooks R.M., Simanek E.E. (2003). Synthesis and characterization of covalently linked single-stranded DNA oligonucleotide-dendron conjugates. Bioconj. Chem..

[36] Umali A.P., Simanek E.E. (2003). Thiol-disulfide exchange yields multivalent dendrimers of melamine. Org. Lett..

[37] Lim J., Venditto V.J., Simanek E.E. (2010). Synthesis and characterization of a triazine dendrimer that sequesters iron(III) using 12 desferrioxamine B groups. Bioorg. Med. Chem..

[38] Sreeperumbuduru R.S., Abid Z.M., Claunch K.M., Chen H.-H., McGillivray S.M., Simanek E.E. (2016). Synthesis and antimicrobial activity of triazine dendrimers with DABCO groups. RSC Adv..

[39] Lalwani S., Chouai A., Perez L.M., Santiago V., Shaunak S., Simanek E.E. (2009). Mimicking PAMAM Dendrimers with Amphoteric, Hybrid Triazine Dendrimers: A Comparison of Dispersity and Stability. Macromolecules.

[40] Lalwani S., Venditto V.J., Chouai A., Rivera G.E., Shaunak S., Simanek E.E. (2009). Electrophoretic Behavior of Anionic Triazine and PAMAM Dendrimers: Methods for Improving Resolution and Assessing Purity Using Capillary Electrophoresis. Macromolecules.

[41] Barata T., Teo I., Lalwani S., Simanek E., Zloh M., Shaunak S. (2011). Computational design principles for bioactive dendrimer constructs as antagonists of the TLR-MD-2-LPS complex. Biomaterials.

[42] Lim J., Simanek E.E. (2008). Synthesis of water-soluble dendrimers based on melamine bearing 16 paclitaxel groups. Org. Lett..

[43] Lo S.-T., Stern S., Clogston J.D., Zheng J., Adiseshaiah P.P., Dobrovolskaia M., Lim J., Patri A.K., Sun X., Simanek E.E. (2010). Biological Assessment of Triazine Dendrimer: Toxicological Profiles, Solution Behavior, Biodistribution, Drug Release and Efficacy in a PEGylated, Paclitaxel Construct. Mol. Pharm..

[44] Lim J., Chouai A., Lo S.T., Liu W., Sun X., Simanek E.E. (2009). Design, Synthesis, Characterization, and Biological Evaluation of Triazine Dendrimers Bearing Paclitaxel Using Ester and Ester/Disulfide Linkages. Bioconj. Chem..

[45] Lim J., Lo S.T., Hill S., Pavan G.M., Sun X., Simanek E.E. (2012). Antitumor Activity and Molecular Dynamics Simulations of Paclitaxel-Laden Triazine Dendrimers. Mol. Pharm..

[46] Lee C., Lo S.T., Lim J., Da Costa V.C., Ramezani S., Öz O.K., Pavan G.M., Annunziata O., Sun X., Simanek E.E. (2013). Design, Synthesis and Biological Assessment of a Triazine Dendrimer with Approximately 16 Paclitaxel Groups and 8 PEG Groups. Mol. Pharm..

[47] Lim J., Guan B., Nham K., Hao G., Sun X., Simanek E.E. (2019). Tumor Uptake of Triazine Dendrimers Decorated with Four, Sixteen, and Sixty-Four PSMA-Targeted Ligands: Passive versus Active Tumor Targeting. Biomolec..

[48] Lim J., Guo Y., Rostollan C.L., Stanfield J., Hsieh J.T., Sun X., Simanek E.E. (2008). The role of the size and number of polyethylene glycol chains in the biodistribution and tumor localization of triazine dendrimers. Mol. Pharm..

[49] Lim J., Turkbey B., Bernardo M., Bryant LHJr Garzoni M., Pavan G.M., Nakajima T., Choyke P.L., Simanek E.E., Kobayashi H. (2012). Gadolinium MRI Contrast Agents Based on Triazine Dendrimers: Relaxivity and In Vivo Pharmacokinetics. Bioconj. Chem..

[50] Lee C., Ji K., Simanek E.E. (2016). Functionalization of a Triazine Dendrimer Presenting Four Maleimides on the Periphery and a DOTA Group at the Core. Molecules.

[51] Neerman M.F., Zhang W., Parrish A.R., Simanek E.E. (2004). In vitro and in vivo evaluation of a melamine dendrimer as a vehicle for drug delivery. Int. J. Pharm..

[52] Enciso A.E., Neun B., Rodriguez J., Ranjan A.P., Dobrovolskaia M.A., Simanek E.E. (2016). Nanoparticle Effects on Human Platelets in Vitro: A Comparison between PAMAM and Triazine Dendrimers. Molecules.

[53] Ilinskaya A.N., Shah A., Enciso A.E., Chan K.C., Kaczmarczyk J.A., Blonder J., Simanek E.E., Dobrovolskaia M.A. (2019). Nanoparticle physicochemical properties determine the activation of intracellular complement. Nanomed Nanotech. Biol. Med..

[54] Neerman M.F., Umali A.P., Chen H.T., Waghela S.D., Parrish A.R., EESimanek E.E. (2005). Biological evaluation of dendrimers based on melamine. J. Drug Deliv. Sci. Tech..

[55] Acosta E.J., Carr C.S., Simanek E.E., Shantz D.F. (2004). Engineering nanospaces: Iterative synthesis of melamine-based dendrimers on amine-functionalized SBA-15 leading to complex hybrids with controllable chemistry and porosity. Adv. Mater..

[56] Yoo S., Lunn J.D., Gonzalez S., Ristich J.A., Simanek E.E., Shantz D.F. (2006). Engineering nanospaces: OMS/dendrimer hybrids possessing controllable chemistry and porosity. Chem. Mater..

[57] Javaid A., Gonzalez S.O., Simanek E.E., Ford D.M. (2006). Nanocomposite membranes of chemisorbed and physisorbed molecules on porous alumina for environmentally important separations. J. Membr. Sci..

[58] Yoo S., Yeu S., Sherman R.L., Simanek E.E., Shantz D.F., Ford D.M. (2009). Reverse-selective membranes formed by dendrimers on mesoporous ceramic supports. J. Membr. Sci..

[59] Acosta E.J., Gonzalez S.O., Simanek E.E. (2005). Synthesis, characterization, and application of melamine based dendrimers supported on silica gel. J. Polym. Sci. A.

[60] Gonzalez S.O., Furyk S., Li C., Tichy S.E., Bergbreiter D.E., Simanek E.E. (2004). Latent solid-phase extraction with thermoresponsive soluble polymers. J. Polym. Sci. A.

[61] Acosta E.J., Steffensen M.B., Tichy S.E., Simanek E.E. (2004). Removal of atrazine from water using covalent sequestration. J. Agric. Food Chem..

[62] Hollink E., Tichy S.E., Simanek E.E. (2005). Piperidine-functionalized supports sequester atrazine from solution. Ind. Eng. Chem. Res..

[63] Huang A.Y.T., Patra S., Chen H.T., Kao C.L., Simanek E.E. (2016). Solid-Phase Synthesis of Libraries of Triazine Dendrimers and Orthogonal Staining Methods for Tracking Reactions on Resin. Asian J. Org. Chem..

[64] Acosta E.J., Deng Y.J., White G.N., Dixon J.B., McInnes K.J., Senseman S.A., Frantzen S.A., Simanek E.E. (2003). Dendritic Surfactants show evidence for frustrated intercalation: A new organoclay morphology. Chem. Mat..

[65] Neitsch S.L., McInnes K.J., Senseman S.A., White G.N., Simanek E.E. (2006). Melamine-based organoclay to sequester atrazine. Chemosphere.

[66] Enciso A.E., Doni G., Nifosi R., Palazzesi F., Gonzalez R., Ellsworth A.A., Coffer J.L., Walker A.V., Pavan G.M., Mohamed A.A. (2017). Facile synthesis of stable, water soluble, dendron-coated gold nanoparticles. Nanoscale.

[67] Ramirez-Crescencio F., Enciso A.E., Hasan M., Da Costa V.C.P., Annunziata O., Redón R., Coffer J.L., Simanek E.E. (2016). Thermoregulated Coacervation, Metal-Encapsulation and Nanoparticle Synthesis in Novel Triazine Dendrimers. Molecules.

[68] Redon R., Ramirez-Crescencio F., Gonzalez-Rodriguez R., Coffer J., Simanek E.E. (2020). Ir(0) and Pt(0) nanoparticle-triazine dendrimer composites. J. Coord. Chem..

[69] Cummins B.M., Lim J., Simanek E.E., Pishko M.V., Coté G.L. (2011). Encapsulation of a Concanavalin A/dendrimer glucose sensing assay within microporated poly (ethylene glycol) microspheres. Biomed. Opt. Express.

[70] Simanek E.E., Enciso A.E. (2015). Cationic Triazine Dendrimers: Synthesis, Characterization, and Biological Applications. Cationic Polym. Regen. Med..

[71] Simanek E.E., Enciso A.E., Pavan G.M. (2013). Computational design principles for the discovery of bioactive dendrimers: [s]-triazines and other examples. Expert Opin. Drug Disc..

[72] Lim J., Simanek E.E. (2012). Triazine dendrimers as drug delivery systems: From synthesis to therapy. Adv. Drug Deliv. Rev..

[73] Simanek E.E., Lim J. (2012). Paclitaxel-Triazine Dendrimer Constructs: Efficacy, Toxicity, and Characterization. Multifunctional Nanoparticles for Drug Delivery Applications: Imaging, Targeting, and Delivery.

[74] Simanek E.E., Abdou H., Lalwani S., Lim J., Mintzer M., Venditto V.J., Vittur B. (2010). The 8 year thicket of triazine dendrimers: Strategies, targets and applications. Proc. R. Soc. A.

[75] Crampton H.L., Simanek E.E. (2007). Dendrimers as drug delivery vehicles: Non-covalent interactions of bioactive compounds with dendrimers. Polym. Int..

[76] Steffensen M.B., Hollink E., Kuschel F., Bauer M., Simanek E.E. (2006). Dendrimers based on [1,3,5]-triazines. J. Polym. Sci. A.

[77] Simanek E.E. (2003). Dendrimers based on melamine: Vehicles for drug delivery?. Polym. Drug Deliv. I Part. Drug Carr. ACS Symp. Ser..

[78] Hollink E., Simanek E.E., Bergbreiter D.E. (2005). Strategies for protecting and manipulating triazine derivatives. Tetrahedron Lett..

[79] Bergbreiter D.E., Simanek E.E., Owsik I. (2005). New synthetic methods for the formation of basic, polyvalent, hyperbranched grafts. J. Poly. Sci. A.

[80] Koskela J.E., Liljeström V., Lim J., Simanek E.E., Ras R.H., Priimagi A., Kostiainen M.A. (2014). Light-Fuelled Transport of Large Dendrimers and Proteins. J. Am. Chem. Soc..

[81] Simanek E.E., Gonzalez S.O. (2002). Dendrimers: Branching out of polymer chemistry. J. Chem. Ed..

